# Timing of acute decompensated heart failure in patients with heart failure and mildly reduced ejection fraction

**DOI:** 10.1007/s00380-024-02505-3

**Published:** 2025-01-22

**Authors:** Henning Johann Steffen, Noah Abel, Felix Lau, Alexander Schmitt, Marielen Reinhardt, Muharrem Akin, Thomas Bertsch, Jonas Rusnak, Kathrin Weidner, Michael Behnes, Ibrahim Akin, Tobias Schupp

**Affiliations:** 1 Medical Faculty Mannheim, Department of Cardiology, Angiology, Haemostaseology and Medical Intensive Care, University Medical Centre Mannheim, Heidelberg University, Theodor-Kutzer-Ufer 1-3, 68167 Mannheim, Germany; 2 Department of Cardiology, St. Josef-Hospital, Ruhr-Universität Bochum, 44791 Bochum, Germany; 3 Institute of Clinical Chemistry, Laboratory Medicine and Transfusion Medicine, Nuremberg General Hospital, Paracelsus Medical University, 90419 Nuremberg, Germany; 4 Department of Cardiology, Angiology and Pneumology, University Hospital Heidelberg, 69120 Heidelberg, Germany

**Keywords:** Heart failure with mildly reduced ejection fraction, HFmrEF, Acute decompensated heart failure, ADHF

## Abstract

This study investigates the prognosis of acute decompensated heart failure (ADHF) on admission (i.e., primary ADHF) as compared to ADHF onset during course of hospitalization (i.e., secondary ADHF) in patients hospitalized with heart failure with mildly reduced ejection fraction (HFmrEF). Limited data regarding the prognostic impact of the timing of onset of ADHF is available. Consecutive patients with HFmrEF and ADHF were retrospectively included at one institution from 2016 to 2022. Patients with primary ADHF were compared to patients with secondary ADHF with regard to the primary endpoint all-cause mortality at 30 months. Kaplan–Meier, uni- and multivariable Cox proportional regression analyses were applied for statistics. From a total of 484 patients hospitalized with HFmrEF and ADHF, 67.98% (n = 329) were admitted with primary ADHF. Patients with secondary ADHF had higher rates of concomitant acute myocardial infarction, alongside with a higher extend of coronary artery disease. The risk of all-cause mortality at 30 months was not affected by the timing of ADHF (hazard ratio (HR) = 0.853; 95% confidence interval (CI) 0.653–1.115; p = 0.246). However, patients with primary ADHF were associated with a higher risk of HF-related rehospitalization at 30 months (HR = 2.513; 95% CI 1.555–4.065; p = 0.001), which was still evident after multivariable adjustment (HR = 2.347; 95% CI 1.418–3.883; p = 0.001). The timing of onset of ADHF was not associated with long-term mortality in HFmrEF, however primary ADHF was associated with a higher risk of HF-related rehospitalization.

## Introduction

The characterization of patients with heart failure with mildly reduced ejection fraction (HFmrEF) has gained more importance following their introduction and upgrade within the 2016 and 2021 European guidelines of heart failure (HF) [1, 2]. HFmrEF, which is characterized by a left ventricular ejection fraction (LVEF) between 41 and 49% and has been recognized as a unique subtype of HF, sharing patterns with both HF with reduced (i.e., HFrEF) and HF with preserved LVEF (i.e., HFpEF) [3–7]. Related to the limited number of randomized controlled trials including patients with HFmrEF, guideline-based treatment recommendations in HFmrEF are scarce.

Acute decompensated heart failure (ADHF) encompasses a diverse range of clinical scenarios characterized by the intensification of manifestations and symptoms associated with heart failure (HF). It constitutes the predominant presentation of acute HF. ADHF can manifest either as the initial presentation of HF or as an acute exacerbation in the setting of chronic HF [8, 9]. Although ADHF was recently shown to impair long-term prognosis in HFmrEF [10], data regarding the characteristics and prognostic impact of concerning timing of ADHF, such as ADHF on admission (i.e., primary ADHF) compared to ADHF during hospitalization (i.e., secondary ADHF) remains scarce. Prior studies primarily focused on ADHF in patients with HFrEF and HFpEF, resulting in a lack of comprehensive research including patients with HFmrEF, emphasizing the need for focused research efforts to enhance the understanding and clinical management of ADHF across the range of HF phenotypes.[11–23].

The present study sought to investigate the prognosis of patients with primary versus secondary ADHF, including consecutive patients hospitalized with HFmrEF from 2016 to 2022.

## Methods

### Study patients, design and data collection:

For the present study, patients hospitalized with HFmrEF and ADHF at a tertiary university medical center were included from January 2016 to December 2022, as recently published [24]. The electronic hospital information system facilitated the comprehensive documentation of relevant clinical data related to the index event. This included baseline characteristics, vital signs upon admission, prior medical history, previous medical interventions, duration of the index hospitalization, and intensive care unit (ICU) stay, as well as laboratory values. Furthermore, noninvasive and invasive cardiac diagnostic information, such as echocardiogram results, coronary angiography findings, and data from existing or newly implanted cardiac devices, were systematically recorded. The monitoring extended beyond the index hospitalization to encompass subsequent outpatient clinic visits, echocardiographic assessments, HF-related rehospitalizations, and adverse cardiac events until the end of 2022.

This investigation originated from the "Heart Failure With Mildly Reduced Ejection Fraction Registry" (HARMER), which is a retrospective single-center registry that included consecutively enrolled patients with HFmrEF at the University Medical Center Mannheim (UMM), Germany (clinicaltrials.gov identifier: NCT05603390). Ethical standards were upheld in accordance with the principles of the Declaration of Helsinki and received approval from the Medical Ethics Committee II of the Medical Faculty Mannheim, University of Heidelberg, Germany (ethical approval code: 2022-818).

### Inclusion and exclusion criteria

For the present study, patients hospitalized with HFmrEF and ADHF from 2016 until 2022 were included. Patients without ADHF either at index admission or during index hospitalization, as well as patients under 18 years of age were excluded. The diagnosis of HFmrEF was performed in accordance with the "2021 ESC Guidelines for the Diagnosis and Treatment of Acute and Chronic Heart Failure" [2]. Patients with a LVEF ranging from 41 to 49%, accompained with symptoms and/or signs of HF, were included. The presence of elevated amino-terminal prohormone of brain natriuretic peptide (NT-proBNP) levels and other indicators of structural heart disease strengthened the diagnostic likelihood, they were not essential for confirming HFmrEF. Transthoracic echocardiography, conducted as part of routine clinical care and in accordance with current European guidelines [25], was performed by cardiologists who remained blinded to the final study analysis. ADHF was defined in accordance with European guidelines [2], emphasizing congestion marked by discernible deterioration in clinical signs and/or symptoms of HF necessitating intravenous diuretic therapy [27].

### Risk stratification

For the present study, risk stratification was performed according to the timing of onset of ADHF. Patients with ADHF on admission (i.e., primary ADHF) were compared to patients with ADHF during course of index hospitalization but without ADHF on admission (i.e., secondary ADHF).

### Study endpoints

The primary endpoint was all-cause mortality at 30 months (median follow-up). Secondary endpoints included in-hospital all-cause mortality, all-cause mortality at 12 and 24 months, rehospitalization for worsening heart failure (HF) at 12, 24 and 30 months, cardiac rehospitalization, acute myocardial infarction (AMI), stroke, coronary revascularization, and major adverse cardiac and cerebrovascular events (MACCE) at 30 months. Documentation of all-cause mortality was facilitated through the electronic hospital information system and direct communication with state resident registration offices (i.e., bureau of mortality statistics). From a total of 2,228 patients diagnosed with HFmrEF, 44 individuals were excluded due to a lack of evidence during long-term follow-up (i.e., lost-to-follow-up rate of 1.97%). HF-related hospitalization was defined as the need for readmission due to worsening HF requiring intravenous diuretic therapy. Cardiac rehospitalization refers to rehospitalization due to a primary cardiac condition, such as worsening HF, AMI, coronary revascularization, and symptomatic atrial or ventricular arrhythmias. MACCE was defined as a composite of all-cause mortality, coronary revascularization, non-fatal AMI, and non-fatal stroke.

### Statistical methods

Quantitative data is depicted as the mean ± standard error of the mean (SEM), median and interquartile range (IQR), and ranges, contingent on the data distribution. Statistical comparisons employed the student’s t-test for normally distributed data or the Mann–Whitney U test for nonparametric data. The Kolmogorov–Smirnov test was utilized to assess deviations from a Gaussian distribution. Qualitative data is displayed as absolute and relative frequencies, and statistical comparisons were conducted using the Chi-square test or Fisher’s exact test, as deemed appropriate. Kaplan–Meier analyses were performed comparing the prognosis of primary versus secondary ADHF and univariable hazard ratios (HR) were given together with 95% confidence intervals. The prognostic impact of the onset of ADHF was thereafter investigated within multivariable Cox regression models using the “forward selection” option.

Results of all statistical tests were considered significant for p ≤ 0.05. SPSS (Version 28, IBM, Armonk, New York) was used for statistics.

## Results

### Study population

**Fig. 1:**
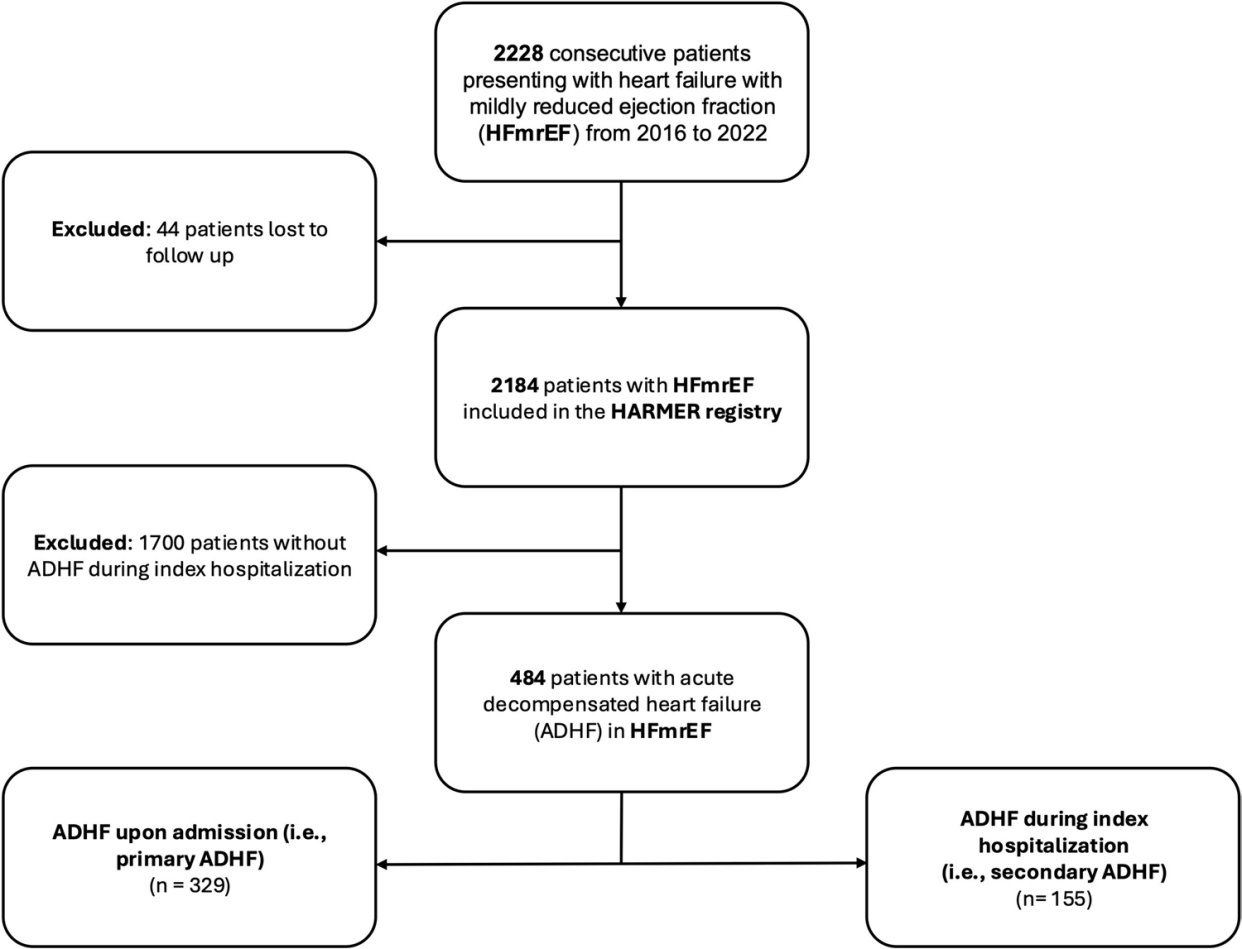
Flow chart of the study population

**Fig. 2:**
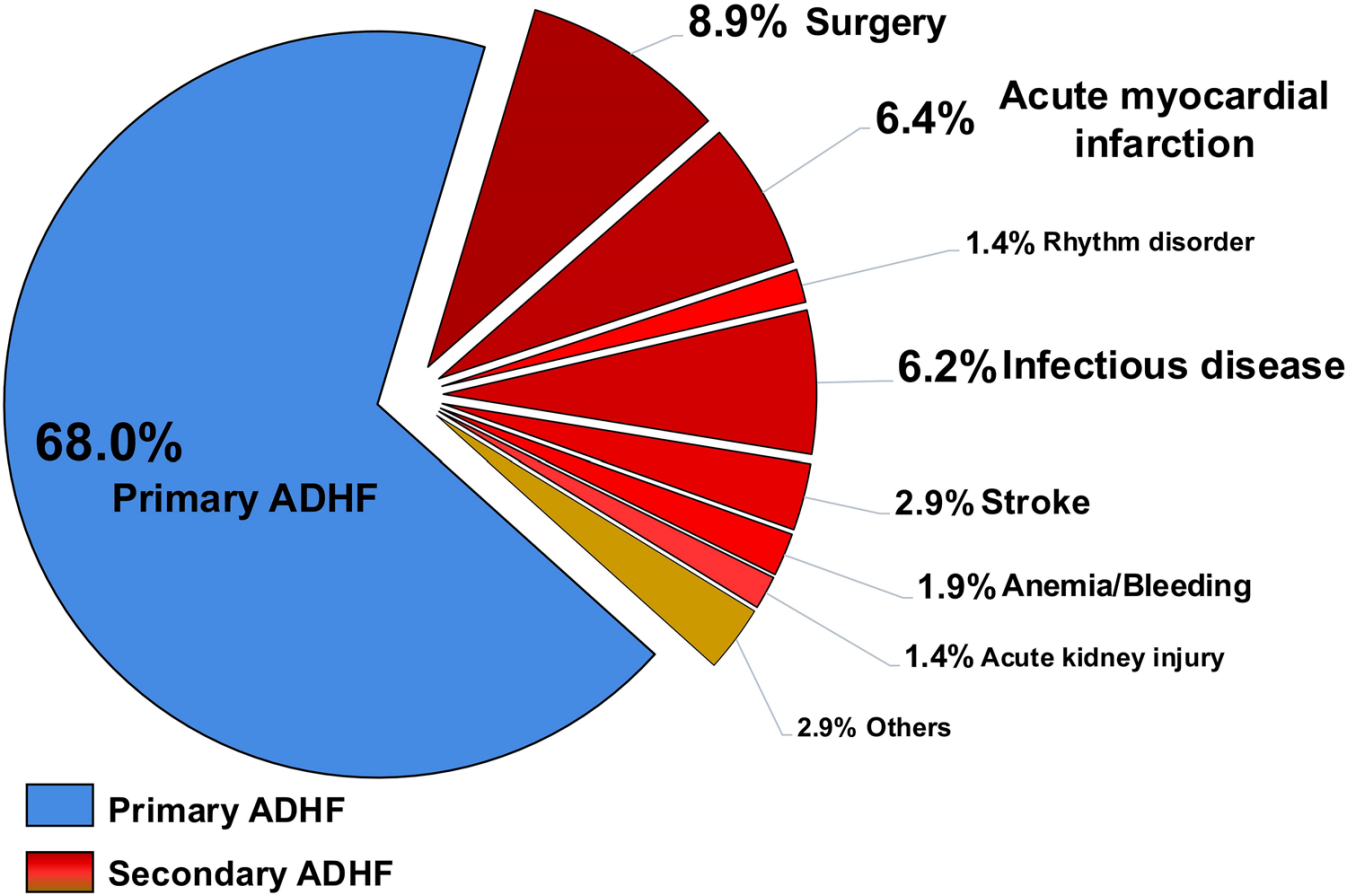
Causes for heart failure in HFmrEF patients

From 2016 to 2022, a total of 2,228 patients with HFmrEF were hospitalized at our institution. Of those, 44 patients lost-to-follow up and 1,700 patients without ADHF during index hospitalization were excluded. Finally, 484 patients with ADHF and HFmrEF were included, of those 329 patients (67.98%) presented with ADHF on admission (i.e., primary ADHF) and 155 patients (32.02%) developed ADHF during course of index hospitalization (i.e., secondary ADHF) (Fig. 1; Flow chart). In patients developing ADHF during index hospitalization (i.e., secondary ADHF), most patients were hospitalized for surgery (8.9%, n = 43), AMI (6.4%, n = 31), and infectious disease (6.2%, n = 30). Other less frequent causes were stroke (2.9%, n = 14), anemia/bleeding (1.9%, n = 9), rhythm disorder (1.4%, n = 7) and acute kidney injury (1.4%, n = 7) (Fig. 2).

As shown in Table 1, patients with primary ADHF had a higher body mass index (BMI) (median 27 kg/m^2^ vs. 25 kg/m^2^; p = 0.002) compared to patients with secondary ADHF. Furthermore, patients with primary ADHF had higher rates of congestive HF (52.6% vs. 41.9%; p = 0.029), chronic kidney disease (CKD) (56.8% vs. 45.8%; p = 0.023) and arterial hypertension (90.3% vs. 77.4%; p = 0.001). Conversely, peripheral artery disease was more common in patients with secondary ADHF (12.5% vs. 21.3%; p = 0.012). With regard to comorbidities during index hospitalization, higher rates of acute coronary syndromes, both ST-segment elevation myocardial infarction (STEMI) (0.9% vs. 9.7%; p = 0.001) and non-ST-segment elevation myocardial infarction (NSTEMI) (5.8% vs. 23.2%; p = 0.001) were observed in patients with secondary ADHF, accompanied by higher rates of cardiopulmonary resuscitation (1.2% vs. 7.7%; p = 0.001).

**Table 1:** Baseline characteristics

	Primary ADHF (*n* = 329)		Secondary ADHF (*n* = 155)		p value
**Age**, median (IQR)	80	(72–86)	80	72–85)	0.610
**Male sex**, n (%)	186	(56.5)	83	(53.5)	0.537
**Body mass index**, kg/m2, median (IQR)	27	(23–33)	25	(23-29)	**0.002**
**SBP**, mmHg, median (IQR)	141	(124–161)	137	(120–156)	0.054
**DBP**, mmHg, median (IQR)	77	(65–90)	70	(62–83)	**0.010**
**Heart rate**, bpm, median (IQR)	84	(70–99)	81	(68–99)	0.363
**Medical history**, n(%)					
Coronary artery disease	149	(45.3)	66	(42.6)	0.576
Prior myocardial infarction	84	(25.5)	39	(25.2)	0.930
Prior PCI	106	(32.2)	43	(27.7)	0.319
Prior CABG	40	(12.2)	14	(9.0)	0.308
Prior valvular surgery	18	(5.5)	4	(2.6)	0.154
Congestive heart failure	173	(52.6)	65	(41.9)	**0.029**
Prior HFrEF	17	(5.2)	7	(4.5)	1.000
Prior HFmrEF	24	(7.3)	16	(10.3)	0.160
Prior HFpEF	65	(19.8)	23	(14.8)	0.214
Prior LVEF not documented	67	(20.3)	19	(12.3)	–
Decompensated heart failure < 12 months	75	(22.8)	26	(16.8)	0.128
Prior ICD	5	(1.5)	1	(0.6)	0.417
Prior sICD	1	(0.3)	0	(0.0)	0.492
Prior CRT-D	7	(2.1)	2	(1.3)	0.525
Prior Pacemaker	49	(14.9)	16	(10.3)	0.169
Chronic kidney disease	187	(56.8)	71	(45.8)	**0.023**
Peripheral artery disease	41	(12.5)	33	(21.3)	**0.012**
Stroke	53	(16.1)	30	(19.4)	0.377
Liver cirrhosis	13	(4.0)	3	(1.9)	0.247
Malignancy	42	(12.8)	28	(18.1)	0.122
COPD	55	(16.7)	20	(12.9)	0.279
**Cardiovascular risk factors**, n (%)					
Arterial hypertension	297	(90.3)	120	(77.4)	**0.001**
Diabetes mellitus	164	(49.8)	67	(43.2)	0.174
Hyperlipidemia	111	(33.7)	48	(31.0)	0.545
Smoking					
Current	32	(9.7)	24	(15.5)	0.065
Former	68	(20.7)	25	(16.1)	0.237
Family history	26	(7.9)	9	(5.8)	0.406
**Comorbidities during index hospitalization**, n (%)					
Acute coronary syndrome					
Unstable angina	11	(3.3)	2	(1.3)	0.192
STEMI	3	(0.9)	15	(9.7)	**0.001**
NSTEMI	19	(5.8)	36	(23.2)	**0.001**
Cardiogenic Shock	7	(2.1)	18	(11.6)	**0.001**
Atrial fibrillation	204	(62.0)	82	(52.9)	0.052
Cardiopulmonary resuscitation	4	(1.2)	12	(7.7)	**0.001**
Out-of-hospital	0	(0.0)	6	(3.9)	**0.001**
In-hospital	4	(1.2)	6	(3.9)	0.055
Stroke	5	(1.5)	11	(7.1)	**0.001**
**Medication on admission**, n (%)					
ACE-inhibitor	138	(41.9)	52	(33.5)	0.078
ARB	86	(26.1)	31	(20.0)	0.141
Beta-blocker	234	(71.1)	98	(63.2)	0.081
Aldosterone antagonist	47	(14.3)	14	(9.0)	0.104
ARNI	3	(0.9)	2	(1.3)	0.701
SGLT2-inhibitor	7	(2.1)	2	(1.3)	0.525
Loop diuretics	230	(69.9)	68	(43.9)	**0.001**
Statin	169	(51.4)	70	(45.2)	0.203
ASA	93	(28.3)	59	(38.1)	**0.030**
P2Y12-inhibitor	36	(10.9)	13	(8.4)	0.385
DOAC	124	(37.7)	39	(25.2)	**0.007**
Vitamin K antagonist	37	(11.2)	13	(8.4)	0.335

Level of significance p ≤ 0.05. Bold type indicates statistical significance

*ACE* angiotensin-converting-enzyme, *ARB* angiotensin receptor blocker, *ARNI* angiotensin receptor neprilysin inhibitor, *ASA* acetylsalicylic acid, *CABG* coronary artery bypass grafting, *CKD* chronic kidney disease, *COPD* chronic obstructive pulmonary disease, *CRT-D* cardiac resynchronization therapy with defibrillator, *DBP* diastolic blood pressure, *DOAC* directly acting oral anticoagulant, *HFmrEF* heart failure with mildly reduced ejection fraction, *HFpEF* heart failure with preserved ejection fraction, *HFrEF* heart failure with reduced ejection fraction, *IQR* interquartile range, *LVEF* left ventricular ejection fraction, *(N)STEMI* non-ST-segment elevation myocardial infarction, *SBP* systolic blood pressure, *SGLT2* sodium glucose linked transporter 2, *(s) ICD* (subcutaneous) implantable cardioverter defibrillator

An ischemic etiology was the most commonly associated with HF in both patients with primary or secondary ADHF (55% vs. 64.5%; p = 0.058) (Table 2). However, patients with primary ADHF presented an increased burden of HF-related symptoms, reflected by a higher NYHA functional class (NYHA III: 45.9% vs. 34.8%; NYHA IV: 32.5% vs. 26.5%; p = 0.001). While there was no statistically significant disparity in the frequency of coronary angiography between the groups, patients with primary ADHF had a higher prevalence of multi-vessel coronary artery disease (CAD) (p = 0.004) but did not undergo more frequent percutaneous coronary interventions (PCI) during index hospitalization (34.2% vs. 58.2%; p = 0.002). With regard to laboratory parameters, patients with primary ADHF had higher creatinine (median 1.40 mg/dl vs. 1.12 mg/dl; p = 0.005) and hemoglobin levels (median 11.4 g/dl vs. 10.6 g/dl; p = 0.001), whereas white blood cell (WBC) count (median 8.08 × 10^9^/L vs. 8.77 × 10^9^/L; p = 0.034), C-reactive protein (CRP; median 18 mmol/L vs. 38 mmol/L; p = 0.001) and NT-proBNP levels were lower compared to patients with secondary ADHF (median 5164 pg/mL vs. 7644 pg/mL; p = 0.024). Ultimately, the prescription rates of angiotensin receptor blockers (ARB; 30.6% vs. 17.4%; p = 0.003) and loop diuretics (93.0% vs. 83.3%; p = 0.002) were higher in patients with primary ADHF.

**Table 2:** Heart-failure related and procedural data

	Primary ADHF (*n* = 329)		Secondary ADHF (*n* = 155)		p value
Heart failure etiology, n (%)					
Ischemic cardiomyopathy	181	(55.0)	100	(64.5)	
Non-ischemic cardiomyopathy	20	(6.1)	11	(7.1)	
Hypertensive cardiomyopathy	26	(7.9)	5	(3.2)	
Congenital heart disease	1	(0.3)	0	(0.0)	
Valvular heart disease	30	(9.1)	6	(3.9)	0.058
Tachycardia associated	20	(6.1)	9	(5.8)	
Tachymyopathy	10	(3.0)	2	(1.3)	
Pacemaker-induced cardiomyopathy	6	(1.8)	0	(0.0)	
Unknown	35	(10.6)	22	(14.2)	
NYHA functional class, n (%)					
I/II	71	(21.6)	60	(38.7)	
III	151	(45.9)	54	(34.8)	**0.001**
IV	107	(32.5)	41	(26.5)	
Echocardiographic data					
LVEF, %, median (IQR)	45	(45–47)	45	(44–47)	0.857
IVSd, median (IQR)	12	(11–14)	12	(10–13)	**0.007**
LVEDD, mm, median (IQR)	50	(46–55)	47	(43–52)	**0.001**
TAPSE, mm, median (IQR)	20	(16–22)	19	(16–23)	0.769
LA diameter, mm, median (IQR)	46	(40–51)	44	(37–47)	**0.005**
LA surface, cm^2^, median (IQR)	25	(21–30)	23	(20–27)	**0.015**
E/A, median (IQR)	1.0	(0.7–1.6)	0.9	(0.6–1.3)	0.137
E/E`, median (IQR)	12.7	(7.5–18.5)	12.0	(7.0–16.0)	0.142
Diastolic dysfunction, n (%)	249	(75.7)	110	(71.0)	0.269
Moderate-severe aortic stenosis, n (%)	48	(14.6)	25	(16.1)	0.659
Moderate-severe aortic regurgitation, n (%)	28	(8.5)	11	(7.1)	0.594
Moderate-severe mitral regurgitation, n (%)	80	(24.3)	36	(23.2)	0.793
Moderate-severe tricuspid regurgitation, n (%)	99	(30.1)	52	(33.5)	0.444
TR Vmax, m/s, median (IQR)	3.10	(2.70–3.40)	3.00	(2.70–3.40)	0.889
VCI, mm, median (IQR)	24	(19–28)	22	(18–26)	0.386
Aortic root, mm, median (IQR)	32	(29–35)	33	(28–36)	0.614
Coronary angiography, n (%)	114	(34.7)	67	(43.2)	0.069
No evidence of coronary artery disease	32	(28.1)	10	(14.9)	
1-vessel disease	24	(21.1)	8	(11.9)	
2-vessel disease	15	(13.2)	5	(7.5)	**0.004**
3-vessel disease	43	(37.7)	44	(65.7)	
CABG	10	(8.8)	5	(7.5)	0.758
Chronic total occlusion	13	(11.4)	8	(11.9)	0.913
PCI, n (%)	39	(34.2)	39	(58.2)	**0.002**
Sent to CABG, n (%)	6	(5.3)	6	(9.0)	0.335
Baseline laboratory values, median (IQR)					
Potassium, mmol/L	3.8	(3.5–4.2)	3.8	(3.5–4.2)	0.537
Sodium, mmol/L	139	(137–142)	139	(137–142)	0.991
Creatinine, mg/dl	1.40	(1.04–1.98)	1.12	(0.87–1.88)	**0.005**
eGFR, mL/min/1.73 ^2^	46	(31–65)	55	(30–79)	**0.006**
Hemoglobin, g/dL	11.4	(9.7–13.2)	10.6	(9.3–11.8)	**0.001**
WBC count, × 10^9^/L	8.08	(6.35–10.21)	8.77	(6.64–11.81)	**0.034**
Platelet count, × 10^9^/L	233	(175–288)	231	(176–295)	0.499
HbA1c, %	6.2	(5.7–7.6)	6.0	(5.5–6.9)	0.175
LDL-cholesterol, mg/dl	86	(62–112)	85	(66–119)	0.488
HDL-cholesterol, mg/dl	42	(35–54)	40	(29–47)	**0.014**
C-reactive protein, mg/L	18	(6–47)	38	(15–91)	**0.001**
NT-proBNP, pg/mL	5164	(2377–10,102)	7644	(2812–16,506)	**0.024**
NT-proBNP (eGFR corrected), pg/mL	2311	(1275–4196)	3608	(1605–8091)	**0.002**
Cardiac troponin I, µg/L	0.03	(0.02–0.10)	0.12	(0.02–1.36)	**0.001**
Medication at discharge, n (%)					
ACE-inhibitor	147	(46.8)	66	(47.8)	0.843
ARB	96	(30.6)	24	(17.4)	**0.003**
Beta-blocker	265	(84.4)	120	(87.0)	0.480
Aldosterone antagonist	79	(25.2)	25	(18.1)	0.101
ARNI	3	(1.0)	4	(2.9)	0.123
SGLT2-inhibitor	15	(4.8)	2	(1.4)	0.087
Loop diuretics	292	(93.0)	115	(83.3)	**0.002**
Statin	200	(63.7)	82	(59.4)	0.388
Digitalis	22	(7.0)	7	(5.1)	0.440
Amiodarone	14	(4.5)	5	(3.6)	0.684
ASA	111	(35.4)	73	(52.9)	**0.001**
P2Y12-inhibitor	74	(23.6)	50	(36.2)	**0.005**
DOAC	150	(47.8)	49	(35.5)	**0.016**
Vitamin K antagonist	33	(10.5)	8	(5.8)	0.108

*ACE* angiotensin-converting enzyme, *ARB* angiotensin receptor blocker, *ARNI* angiotensin receptor neprilysin inhibitor, *ASA* acetylsalicylic acid, *CABG* coronary artery bypass grafting, *COPD* chronic obstructive pulmonary disease, *DOAC* directly acting oral anticoagulant, *eGFR* estimated glomerular filtration rate, *HbA1c* glycated haemoglobin, *HDL* high-density lipoprotein, *IQR* interquartile range, *IVSd* Interventricular septal end diastole, *LA* left atrial, *LDL* low-density lipoprotein, *LVEDD* Left ventricular end-diastolic diameter, *LVEF* left ventricular ejection fraction, *NT-pro BNP* aminoterminal pro-B-type natriuretic peptide, *NYHA* New York Heart Association, *PCI* percutaneous coronary intervention, *SGLT2* sodium glucose linked transporter 2, *TAPSE* tricuspid annular plane systolic excursion, *VCI* Vena cava inferior, *WBC* white blood cells. Level of significance p ≤ 0.05. Bold type indicates statistical significance

### Prognosis of primary versus secondary ADHF in HFmrEF

**Fig. 3:**
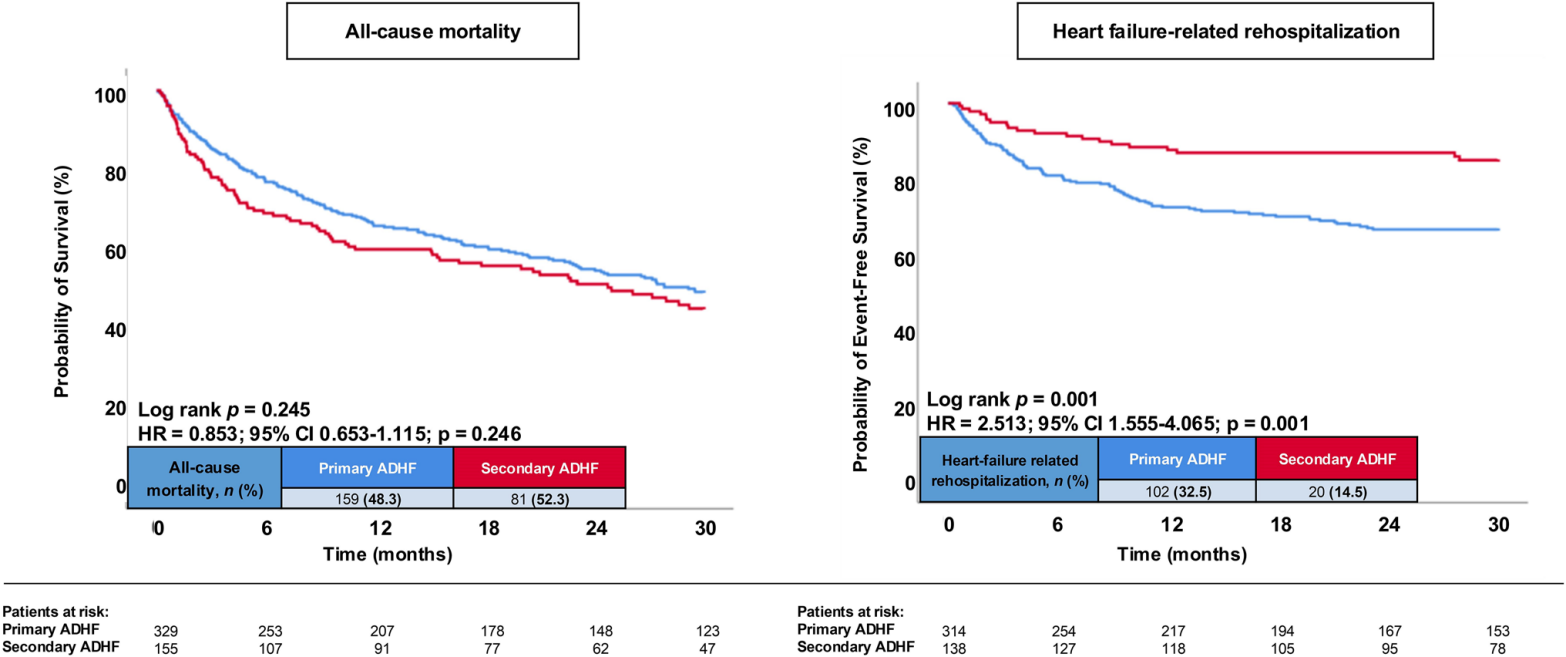
Kaplan–Meier curves showing the probability of freedom from all-cause mortality (left panel) and freedom from HF-related rehospitalization (right panel) at 30 months in patients with HFmrEF and primary vs. secondary ADHF

At 30 months, the primary endpoint of all-cause mortality occured in 48.3% of patients with primary ADHF and in 52.3% with secondary ADHF (Fig. 3; left panel; Table 3). The timing of onset of ADHF was not associated with the risk of 30-months all-cause mortality in HFmrEF (HR = 0.853; 95% CI 0.653–1.115; p = 0.246). In contrast, primary ADHF was associated with a higher risk of HF-related rehospitalization (32.5% vs. 14.5%; HR = 2.513; 95% CI 1.555–4.065; p = 0.001) (Fig. 3; right panel; Table 3). This was already observed after 12 or 24 months of follow-up. In line, primary ADHF was associated with a higher risk of cardiac rehospitalization (38.5% vs. 16.7% HR = 2.644; 95% CI 1.693–4.131; p = 0.001).

**Table 3:** Follow-up data, primary and secondary endpoints

	Primary ADHF (*n* = 329)	Secondary ADHF (*n* = 155)	HR	95% CI	p value
Primary endpoints, n (%)					
All-cause mortality, at 30 months	159 (48.3)	81 (52.3)	0.853	0.653–1.115	0.246
Secondary endpoints, n (%)					
All-cause mortality, at 12 months	112 (34.0)	62 (40.0)	0.792	0.580–1.080	0.140
All-cause mortality, at 24 months	144 (43.8)	74 (47.7)	0.852	0.644–1.127	0.262
Heart-failure related rehospitalization, at 12 months	86 (27.4)	17 (12.3)	2.439	1.449–4.098	**0.001**
Heart-failure related rehospitalization, at 24 months	102 (32.5)	18 (13.0)	2.785	1.689–4.608	**0.001**
Heart-failure related rehospitalization, at 30 months	102 (32.5)	20 (14.5)	2.513	1.555–4.065	**0.001**
Cardiac rehospitalization, at 30 months	121 (38.5)	23 (16.7)	2.644	1.693–4.131	**0.001**
Coronary revascularization, at 30 months	17 (5.4)	4 (2.9)	1.892	0.637–5.622	0.251
Acute myocardial infarction, at 30 months	12 (3.8)	1 (0.7)	5.370	0.698–41.298	0.106
Stroke, at 30 months	11 (3.5)	3 (2.2)	1.631	0.455–5.847	0.453
MACCE, at 30 months	172 (52.3)	86 (55.5)	0.891	0.698–1.155	0.385
Follow-up data, median (IQR)					
Hospitalization time, days	11 (8–18)	21 (14–35)	**0.001**		–
ICU time, days	0 (0–0)	0 (0–3)	**0.001**		–
Follow-up time, days	632 (219–1225)	536 (116–1188)	0.114		–

*CI* confidence interval, *COPD* chronic obstructive pulmonary disease, *HR* hazard ratio, *ICU* intensive care unit, *IQR* interquartile range, *MACCE* major adverse cardiac and cerebrovascular events. Level of significance p ≤ 0.05. Bold type indicates statistical significance

Further secondary endpoints, including the risks of all-cause mortality at 12 months, coronary revascularization and AMI at 30 months did not significantly differ in patients with primary or secondary ADHF (Table 3).

### Multivariable risk adjustment

After adjustment for patients’ characteristics and comorbidities, patients with primary and secondary ADHF were associated with comparable risk of 30-months all-cause mortality (HR = 0.943; 95% CI 0.696–1.278; p = 0.706) (Table 4; left panel). Advanced age (HR = 1.039; 95% CI 1.023–1.055; p = 0.001; per year increase), a history of congestive HF (HR = 1.453; 95% CI 1.061–1.990; p = 0.020), prior CKD (HR = 1.454; 95% CI 1.080–1.958; p = 0.014), prior AMI (HR = 1.804; 95% CI 1.194–2.725; p = 0.005), tricuspid annular plane systolic excursion (TAPSE) < 18 mm (HR = 1.450; 95% CI 1.105–1.903; p = 0.007) and anemia (HR = 1.540; 95% CI 1.120–2.117; p = 0.008) were identified to increase the risk of 30-month all-cause mortality (Table 4; left panel).

**Table 4:** Multivariable Cox regression analyses with regard to 30-months all-cause mortality and heart failure-related re-hospitalization

Variables	All-cause mortality			Heart failure-related re-hospitalization		
	HR	95% CI	p value	HR	95% CI	p value
Age (per year increase)	1.039	1.023–1.055	**0.001**	0.994	0.975–1.014	0.582
Male sex	1.237	0.941–1.627	0.127	0.857	0.593–1.238	0.411
Prior congestive heart failure	1.453	1.061–1.990	**0.020**	1.430	0.912–2.241	0.119
Prior decompensated heart failure < 12 months	1.034	0.729–1.468	0.850	1.310	0.836–2.052	0.238
Chronic kidney disease	1.454	1.080–1.958	**0.014**	1.488	0.987–2.243	0.058
Diabetes mellitus	0.858	0.658–1.118	0.257	1.082	0.748–1.566	0.674
Acute myocardial infarction	1.804	1.194–2.725	**0.005**	0.803	0.397–1.625	0.542
Atrial fibrillation	1.109	0.827–1.488	0.489	1.865	1.191–2.921	**0.006**
Ischemic cardiomyopathy	0.690	0.515–0.923	**0.012**	1.587	1.064–2.369	**0.024**
TAPSE < 18mm	1.450	1.105–1.903	**0.007**	1.032	0.701–1.518	0.874
Anemia	1.540	1.120–2.117	**0.008**	1.337	0.875–2.043	0.180
Primary vs. secondary ADHF	0.943	0.696–1.278	0.706	2.347	1.418–3.883	**0.001**

*ADHF* acute decompensated heart failure, *CI* confidence interval, *HR* adjusted hazard ratio, *TAPSE* tricuspid annular plane systolic excursion. Level of significance p ≤ 0.05. Bold type indicates statistical significance

However, the risk of HF-related rehospitalization at 30-month was higher in patients with primary ADHF (HR = 2.347; 95% CI 1.418–3.883; p = 0.001), even after multivariable adjustment (Table 4; right panel).

## Discussion

The aim of the study was to investigate the prognostic impact of the timing of ADHF (i.e., primary ADHF or secondary ADHF) in patients hospitalized with HFmrEF. Patients with secondary ADHF were admitted with higher rates of concomitant AMI, alongside with a higher rate of multi-vessel CAD. The timing of ADHF was not associated with the risk of all-cause mortality in patients with HFmrEF. However, primary ADHF was associated with an increased risk of rehospitalization for worsening HF, which was still evident after multivariable adjustment.

ADHF is linked to high mortality, frequent hospitalizations, and reduced quality of life, resulting in a substantial economic burden and complications in patient care [13]. Effective management and early intervention are crucial to improving outcomes in patients admitted with ADHF. Although ADHF is a major complication of HF, data investigating the prognosis of patients with ADHF is mainly derived from studies on HFrEF or HFpEF. Recently, our study group demonstrated ADHF is common, affecting 22% of consecutive patients hospitalized with HFmrEF and independently associated with impaired long-term prognosis [10]. The incidence of ADHF was even higher in a study by Farmakis et al*.* including 811 patients with HFmrEF (corresponding incidence of ADHF: 24.9%) [28]. Although ADHF was shown to be an independent risk factor of both all-cause mortality and recurrent hospitalization for worsening HF, the prognostic impact of the timing of ADHF has rarely been investigated.

Characteristics of patients with primary and secondary ADHF were shown to differ significantly within the present study, including higher rates of concomitant CKD and prior congestive HF in patients with primary ADHF, as well as increased rates of concomitant AMI, multi-vessel CAD and stroke in patients with secondary ADHF, whereas the risk of long-term all-cause mortality was not affected by the timing of ADHF. This disparity likely reflects differences in the underlying pathophysiology. Primary ADHF may predominantly driven by intrinsic disease progression, whereas secondary ADHF is often triggered by transient factors such as infections, arrhythmias, or fluid overload [29, 30]. The comorbidity burden in primary ADHF is a well-recognized risk factor for recurrent hospitalizations, as shown in studies linking CKD, prior HF, and multi-morbidity to poorer outcomes [31]. Patel et al. [30] confirm that patients with post-admission (i.e. secondary) ADHF exhibit a wide range of precipitating factors for ADHF, including circulatory issues, digestive abnormalities, or infections. These transient triggers, when effectively managed, may result in better long-term outcomes, as highlighted in studies emphasizing the impact of targeted interventions on secondary ADHF outcomes [32]. The presumed pathomechanism may involve the administration of intravenous fluids, such as in the context of concomitant gastrointestinal bleeding, AMI with subsequent intervention, specifically in patients with concomitant renal failure. Plant et al*.* [33] also highlight that intravenous fluid administration is the second most common reason (23.8% of secondary ADHF) for the development of ADHF. In their study, the most frequent cause of secondary ADHF was found to be pulmonary infection. These findings were also underlined by the study of Taylor et al. [34]. In our study, we demonstrated that 32% of patients experienced ADHF during their inpatient stay. Furthermore, we were able to confirm the hypotheses of these previous studies, which suggest that iatrogenic causes are most often responsible for decompensation during hospitalization.

A study by Savarese et al*.* [35] indicated that HFmrEF shares many similarities in clinical presentation and response to HF therapies with HFrEF. Data from the ESC-HF-LT registry [36] revealed that HFmrEF and HFrEF patients share similar baseline characteristics, such as younger age, higher rates of male sex, ischemic etiology, and lower prevalence of atrial fibrillation. HFmrEF patients had a lower NYHA class, reduced diuretic use, and fewer comorbidities. The CHARM programme [37] supports these findings, showing that HFmrEF patients are like HFrEF patients in terms of age, blood pressure, sex, and history of AMI. From this perspective, the present study may support similarities for patients with HFmrEF and HFrEF, whereas a close follow-up of regarding fluid administration is deemed necessary, specifically with the high rate of patients with secondary ADHF in the present study. In line with this, HFmrEF and HFpEF patients were shown to be associated with a lower risk of cardiovascular events compared to HFrEF, with similar overall survival rates and all-cause mortality between HFmrEF and HFrEF.

This may be further supported by recent studies demonstrating specifically inhibitors of the renin–angiotensin–aldosterone system were shown to be effective for both patients with HFrEF and HFmrEF, but not for HFpEF [38]. Although there are no dedicated intervention studies for HFmrEF, many patients with this condition receive HFrEF therapies. Diuretics are commonly used to alleviate symptoms in HFmrEF patients [1, 4]. In line, the CHARM-preserved trial [39] demonstrated that candesartan reduced the risk of cardiovascular death and rehospitalization in both HFmrEF and HFrEF patients, while beta-blockers lower all-cause and cardiovascular mortality in those with sinus rhythm [40]. From this perspective, the prescription rates of ARBs were significantly lower in patients with secondary ADHF, which were recently shown to be associate with improved long-term all-cause mortality in HFmrEF [41]. Tailored strategies focusing on both pharmacological therapies, such as ARBs and beta-blockers, and structured follow-up programs have been shown to improve long-term outcomes in patients with HFmrEF and HFrEF [26, 39].

It may therefore be hypothesized whether higher prescription rates may have led to improved long-term prognosis, specifically in patients with secondary ADHF. Given the overall poor prognosis in patients with HFmrEF and ADHF, the present study underlines the importance of guideline-remanded HF pharmacotherapies, even in patients with secondary ADHF.

### Clinical implications

Our study provides valuable insights into the clinical management of patients with HFmrEF and ADHF. A substantial long-term all-cause mortality risk among patients with ADHF and HFmrEF was observed, with survival rates approximating 50% at 30 months. These data underscore the importance of distinguishing between primary and secondary ADHF to inform targeted treatment strategies. Primary ADHF, predominantly driven by intrinsic disease progression, necessitates comprehensive optimization of GDMT and structured follow-up programs to minimize the risk of recurrent hospitalizations. Conversely, secondary ADHF, often triggered by reversible factors such as infections, acute myocardial infarction, or surgical interventions, requires a focused approach addressing these precipitating factors. Effective management strategies for secondary ADHF during hospitalization include strict fluid control, prompt infection management, and timely implementation of cardiac interventions.

Moreover, our findings highlight the necessity of close clinical follow-up for HFmrEF patients admitted to non-cardiac units (e.g., for surgical or infectious disease management) to reduce the risk of subsequent secondary ADHF events. These results may emphasize the importance of personalized, etiology-specific management strategies to improve long-term outcomes in this high-risk population.

### Limitations

Related to the retrospective and single-center study design, measured and unmeasured confounding factors may still be present despite multivariable adjustment, which may limit the generalizability of the study. HF-related and cardiac rehospitalizations were assessed at our institution only. Information regarding perfusion status and fluid administration was not available for the present study. Finally, causes of death beyond the index hospitalization were not available for the present study.

## Conclusion

In patients hospitalized with HFmrEF, secondary ADHF accounts for almost one third of ADHF episodes. Primary and secondary ADHF were associated with similar risk of 30-month all-cause mortality, however primary ADHF was independently associated with a higher risk of rehospitalization for HF.
