# Correction: Timing of acute decompensated heart failure in patients with heart failure and mildly reduced ejection fraction

**DOI:** 10.1007/s00380-025-02535-5

**Published:** 2025-04-02

**Authors:** Henning Johann Steffen, Noah Abel, Felix Lau, Alexander Schmitt, Marielen Reinhardt, Muharrem Akin, Thomas Bertsch, Jonas Rusnak, Kathrin Weidner, Michael Behnes, Ibrahim Akin, Tobias Schupp

**Affiliations:** 1 Medical Faculty Mannheim, Department of Cardiology, Angiology, Haemostaseology and Medical Intensive Care, University Medical Centre Mannheim, Heidelberg University, Theodor-Kutzer-Ufer 1-3, 68167 Mannheim, Germany; 2 Department of Cardiology, St. Josef-Hospital, Ruhr-Universität Bochum, 44791 Bochum, Germany; 3 Institute of Clinical Chemistry, Laboratory Medicine and Transfusion Medicine, Nuremberg General Hospital, Paracelsus Medical University, 90419 Nuremberg, Germany; 4 Department of Cardiology, Angiology and Pneumology, University Hospital Heidelberg, 69120 Heidelberg, Germany

**Correction: Heart and Vessels** 10.1007/s00380-024-02505-3

In this article, tables 1 and 2 were published incorrectly.

In table 1, the p value 0.052 of Atrial fibrillation and 0.055 of In-hospital were in bold inadvertently. In table 2, the unit of Creatinine, LDL-cholesterol and HDL-cholesterol were in mmol/L which should have been mg/dl.

The original article has been corrected.

The old incorrect and the corrected versions of Tables 1 and 2 are displayed below.

**Incorrect version:**

**Table 1:** Baseline characteristics

	Primary ADHF (*n* = 329)		Secondary ADHF (*n* = 155)		p value
**Age**, median (IQR)	80	(72–86)	80	72–85)	0.610
**Male sex**, n (%)	186	(56.5)	83	(53.5)	0.537
**Body mass index**, kg/m2, median (IQR)	27	(23–33)	25	(23-29)	**0.002**
**SBP**, mmHg, median (IQR)	141	(124–161)	137	(120–156)	0.054
**DBP**, mmHg, median (IQR)	77	(65–90)	70	(62–83)	**0.010**
**Heart rate**, bpm, median (IQR)	84	(70–99)	81	(68–99)	0.363
**Medical history**, n(%)					
Coronary artery disease	149	(45.3)	66	(42.6)	0.576
Prior myocardial infarction	84	(25.5)	39	(25.2)	0.930
Prior PCI	106	(32.2)	43	(27.7)	0.319
Prior CABG	40	(12.2)	14	(9.0)	0.308
Prior valvular surgery	18	(5.5)	4	(2.6)	0.154
Congestive heart failure	173	(52.6)	65	(41.9)	**0.029**
Prior HFrEF	17	(5.2)	7	(4.5)	1.000
Prior HFmrEF	24	(7.3)	16	(10.3)	0.160
Prior HFpEF	65	(19.8)	23	(14.8)	0.214
Prior LVEF not documented	67	(20.3)	19	(12.3)	–
Decompensated heart failure < 12 months	75	(22.8)	26	(16.8)	0.128
Prior ICD	5	(1.5)	1	(0.6)	0.417
Prior sICD	1	(0.3)	0	(0.0)	0.492
Prior CRT-D	7	(2.1)	2	(1.3)	0.525
Prior Pacemaker	49	(14.9)	16	(10.3)	0.169
Chronic kidney disease	187	(56.8)	71	(45.8)	**0.023**
Peripheral artery disease	41	(12.5)	33	(21.3)	**0.012**
Stroke	53	(16.1)	30	(19.4)	0.377
Liver cirrhosis	13	(4.0)	3	(1.9)	0.247
Malignancy	42	(12.8)	28	(18.1)	0.122
COPD	55	(16.7)	20	(12.9)	0.279
**Cardiovascular risk factors**, n (%)					
Arterial hypertension	297	(90.3)	120	(77.4)	**0.001**
Diabetes mellitus	164	(49.8)	67	(43.2)	0.174
Hyperlipidemia	111	(33.7)	48	(31.0)	0.545
Smoking					
Current	32	(9.7)	24	(15.5)	0.065
Former	68	(20.7)	25	(16.1)	0.237
Family history	26	(7.9)	9	(5.8)	0.406
**Comorbidities during index hospitalization**, n (%)					
Acute coronary syndrome					
Unstable angina	11	(3.3)	2	(1.3)	0.192
STEMI	3	(0.9)	15	(9.7)	**0.001**
NSTEMI	19	(5.8)	36	(23.2)	**0.001**
Cardiogenic Shock	7	(2.1)	18	(11.6)	**0.001**
Atrial fibrillation	204	(62.0)	82	(52.9)	**0.052**
Cardiopulmonary resuscitation	4	(1.2)	12	(7.7)	**0.001**
Out-of-hospital	0	(0.0)	6	(3.9)	**0.001**
In-hospital	4	(1.2)	6	(3.9)	**0.055**
Stroke	5	(1.5)	11	(7.1)	**0.001**
**Medication on admission**, n (%)					
ACE-inhibitor	138	(41.9)	52	(33.5)	0.078
ARB	86	(26.1)	31	(20.0)	0.141
Beta-blocker	234	(71.1)	98	(63.2)	0.081
Aldosterone antagonist	47	(14.3)	14	(9.0)	0.104
ARNI	3	(0.9)	2	(1.3)	0.701
SGLT2-inhibitor	7	(2.1)	2	(1.3)	0.525
Loop diuretics	230	(69.9)	68	(43.9)	**0.001**
Statin	169	(51.4)	70	(45.2)	0.203
ASA	93	(28.3)	59	(38.1)	**0.030**
P2Y12-inhibitor	36	(10.9)	13	(8.4)	0.385
DOAC	124	(37.7)	39	(25.2)	**0.007**
Vitamin K antagonist	37	(11.2)	13	(8.4)	0.335

Level of significance p ≤ 0.05. Bold type indicates statistical significance

*ACE* angiotensin-converting-enzyme, *ARB* angiotensin receptor blocker, *ARNI* angiotensin receptor neprilysin inhibitor, *ASA* acetylsalicylic acid, *CABG* coronary artery bypass grafting, *CKD* chronic kidney disease, *COPD* chronic obstructive pulmonary disease, *CRT-D* cardiac resynchronization therapy with defibrillator, *DBP* diastolic blood pressure, *DOAC* directly acting oral anticoagulant, *HFmrEF* heart failure with mildly reduced ejection fraction, *HFpEF* heart failure with preserved ejection fraction, *HFrEF* heart failure with reduced ejection fraction, *IQR* interquartile range, *LVEF* left ventricular ejection fraction, *(N)STEMI* non-ST-segment elevation myocardial infarction, *SBP* systolic blood pressure, *SGLT2* sodium glucose linked transporter 2, *(s) ICD* (subcutaneous) implantable cardioverter defibrillator

**Table 2:** Heart-failure related and procedural data

	Primary ADHF (*n* = 329)		Secondary ADHF (*n* = 155)		p value
Heart failure etiology, n (%)					
Ischemic cardiomyopathy	181	(55.0)	100	(64.5)	
Non-ischemic cardiomyopathy	20	(6.1)	11	(7.1)	
Hypertensive cardiomyopathy	26	(7.9)	5	(3.2)	
Congenital heart disease	1	(0.3)	0	(0.0)	
Valvular heart disease	30	(9.1)	6	(3.9)	0.058
Tachycardia associated	20	(6.1)	9	(5.8)	
Tachymyopathy	10	(3.0)	2	(1.3)	
Pacemaker-induced cardiomyopathy	6	(1.8)	0	(0.0)	
Unknown	35	(10.6)	22	(14.2)	
NYHA functional class, n (%)					
I/II	71	(21.6)	60	(38.7)	
III	151	(45.9)	54	(34.8)	**0.001**
IV	107	(32.5)	41	(26.5)	
Echocardiographic data					
LVEF, %, median (IQR)	45	(45–47)	45	(44–47)	0.857
IVSd, median (IQR)	12	(11–14)	12	(10–13)	**0.007**
LVEDD, mm, median (IQR)	50	(46–55)	47	(43–52)	**0.001**
TAPSE, mm, median (IQR)	20	(16–22)	19	(16–23)	0.769
LA diameter, mm, median (IQR)	46	(40–51)	44	(37–47)	**0.005**
LA surface, cm^2^, median (IQR)	25	(21–30)	23	(20–27)	**0.015**
E/A, median (IQR)	1.0	(0.7–1.6)	0.9	(0.6–1.3)	0.137
E/E`, median (IQR)	12.7	(7.5–18.5)	12.0	(7.0–16.0)	0.142
Diastolic dysfunction, n (%)	249	(75.7)	110	(71.0)	0.269
Moderate-severe aortic stenosis, n (%)	48	(14.6)	25	(16.1)	0.659
Moderate-severe aortic regurgitation, n (%)	28	(8.5)	11	(7.1)	0.594
Moderate-severe mitral regurgitation, n (%)	80	(24.3)	36	(23.2)	0.793
Moderate-severe tricuspid regurgitation, n (%)	99	(30.1)	52	(33.5)	0.444
TR Vmax, m/s, median (IQR)	3.10	(2.70–3.40)	3.00	(2.70–3.40)	0.889
VCI, mm, median (IQR)	24	(19–28)	22	(18–26)	0.386
Aortic root, mm, median (IQR)	32	(29–35)	33	(28–36)	0.614
Coronary angiography, n (%)	114	(34.7)	67	(43.2)	0.069
No evidence of coronary artery disease	32	(28.1)	10	(14.9)	
1-vessel disease	24	(21.1)	8	(11.9)	
2-vessel disease	15	(13.2)	5	(7.5)	**0.004**
3-vessel disease	43	(37.7)	44	(65.7)	
CABG	10	(8.8)	5	(7.5)	0.758
Chronic total occlusion	13	(11.4)	8	(11.9)	0.913
PCI, n (%)	39	(34.2)	39	(58.2)	**0.002**
Sent to CABG, n (%)	6	(5.3)	6	(9.0)	0.335
Baseline laboratory values, median (IQR)					
Potassium, mmol/L	3.8	(3.5–4.2)	3.8	(3.5–4.2)	0.537
Sodium, mmol/L	139	(137–142)	139	(137–142)	0.991
Creatinine, mmol/L	1.40	(1.04–1.98)	1.12	(0.87–1.88)	**0.005**
eGFR, mL/min/1.73 ^2^	46	(31–65)	55	(30–79)	**0.006**
Hemoglobin, g/dL	11.4	(9.7–13.2)	10.6	(9.3–11.8)	**0.001**
WBC count, × 10^9^/L	8.08	(6.35–10.21)	8.77	(6.64–11.81)	**0.034**
Platelet count, × 10^9^/L	233	(175–288)	231	(176–295)	0.499
HbA1c, %	6.2	(5.7–7.6)	6.0	(5.5–6.9)	0.175
LDL-cholesterol, mmol/L	86	(62–112)	85	(66–119)	0.488
HDL-cholesterol, mmol/L	42	(35–54)	40	(29–47)	**0.014**
C-reactive protein, mg/L	18	(6–47)	38	(15–91)	**0.001**
NT-proBNP, pg/mL	5164	(2377–10,102)	7644	(2812–16,506)	**0.024**
NT-proBNP (eGFR corrected), pg/mL	2311	(1275–4196)	3608	(1605–8091)	**0.002**
Cardiac troponin I, µg/L	0.03	(0.02–0.10)	0.12	(0.02–1.36)	**0.001**
Medication at discharge, n (%)					
ACE-inhibitor	147	(46.8)	66	(47.8)	0.843
ARB	96	(30.6)	24	(17.4)	**0.003**
Beta-blocker	265	(84.4)	120	(87.0)	0.480
Aldosterone antagonist	79	(25.2)	25	(18.1)	0.101
ARNI	3	(1.0)	4	(2.9)	0.123
SGLT2-inhibitor	15	(4.8)	2	(1.4)	0.087
Loop diuretics	292	(93.0)	115	(83.3)	**0.002**
Statin	200	(63.7)	82	(59.4)	0.388
Digitalis	22	(7.0)	7	(5.1)	0.440
Amiodarone	14	(4.5)	5	(3.6)	0.684
ASA	111	(35.4)	73	(52.9)	**0.001**
P2Y12-inhibitor	74	(23.6)	50	(36.2)	**0.005**
DOAC	150	(47.8)	49	(35.5)	**0.016**
Vitamin K antagonist	33	(10.5)	8	(5.8)	0.108

*ACE* angiotensin-converting enzyme, *ARB* angiotensin receptor blocker, *ARNI* angiotensin receptor neprilysin inhibitor, *ASA* acetylsalicylic acid, *CABG* coronary artery bypass grafting, *COPD* chronic obstructive pulmonary disease, *DOAC* directly acting oral anticoagulant, *eGFR* estimated glomerular filtration rate, *HbA1c* glycated haemoglobin, *HDL* high-density lipoprotein, *IQR* interquartile range, *IVSd* Interventricular septal end diastole, *LA* left atrial, *LDL* low-density lipoprotein, *LVEDD* Left ventricular end-diastolic diameter, *LVEF* left ventricular ejection fraction, *NT-pro BNP* aminoterminal pro-B-type natriuretic peptide, *NYHA* New York Heart Association, *PCI* percutaneous coronary intervention, *SGLT2* sodium glucose linked transporter 2, *TAPSE* tricuspid annular plane systolic excursion, *VCI* Vena cava inferior, *WBC* white blood cells. Level of significance p ≤ 0.05. Bold type indicates statistical significance

**Correct version:**

**Table 1:** Baseline characteristics

	Primary ADHF (*n* = 329)		Secondary ADHF (*n* = 155)		p value
**Age**, median (IQR)	80	(72–86)	80	72–85)	0.610
**Male sex**, n (%)	186	(56.5)	83	(53.5)	0.537
**Body mass index**, kg/m2, median (IQR)	27	(23–33)	25	(23-29)	**0.002**
**SBP**, mmHg, median (IQR)	141	(124–161)	137	(120–156)	0.054
**DBP**, mmHg, median (IQR)	77	(65–90)	70	(62–83)	**0.010**
**Heart rate**, bpm, median (IQR)	84	(70–99)	81	(68–99)	0.363
**Medical history**, n(%)					
Coronary artery disease	149	(45.3)	66	(42.6)	0.576
Prior myocardial infarction	84	(25.5)	39	(25.2)	0.930
Prior PCI	106	(32.2)	43	(27.7)	0.319
Prior CABG	40	(12.2)	14	(9.0)	0.308
Prior valvular surgery	18	(5.5)	4	(2.6)	0.154
Congestive heart failure	173	(52.6)	65	(41.9)	**0.029**
Prior HFrEF	17	(5.2)	7	(4.5)	1.000
Prior HFmrEF	24	(7.3)	16	(10.3)	0.160
Prior HFpEF	65	(19.8)	23	(14.8)	0.214
Prior LVEF not documented	67	(20.3)	19	(12.3)	–
Decompensated heart failure < 12 months	75	(22.8)	26	(16.8)	0.128
Prior ICD	5	(1.5)	1	(0.6)	0.417
Prior sICD	1	(0.3)	0	(0.0)	0.492
Prior CRT-D	7	(2.1)	2	(1.3)	0.525
Prior Pacemaker	49	(14.9)	16	(10.3)	0.169
Chronic kidney disease	187	(56.8)	71	(45.8)	**0.023**
Peripheral artery disease	41	(12.5)	33	(21.3)	**0.012**
Stroke	53	(16.1)	30	(19.4)	0.377
Liver cirrhosis	13	(4.0)	3	(1.9)	0.247
Malignancy	42	(12.8)	28	(18.1)	0.122
COPD	55	(16.7)	20	(12.9)	0.279
**Cardiovascular risk factors**, n (%)					
Arterial hypertension	297	(90.3)	120	(77.4)	**0.001**
Diabetes mellitus	164	(49.8)	67	(43.2)	0.174
Hyperlipidemia	111	(33.7)	48	(31.0)	0.545
Smoking					
Current	32	(9.7)	24	(15.5)	0.065
Former	68	(20.7)	25	(16.1)	0.237
Family history	26	(7.9)	9	(5.8)	0.406
**Comorbidities during index hospitalization**, n (%)					
Acute coronary syndrome					
Unstable angina	11	(3.3)	2	(1.3)	0.192
STEMI	3	(0.9)	15	(9.7)	**0.001**
NSTEMI	19	(5.8)	36	(23.2)	**0.001**
Cardiogenic Shock	7	(2.1)	18	(11.6)	**0.001**
Atrial fibrillation	204	(62.0)	82	(52.9)	0.052
Cardiopulmonary resuscitation	4	(1.2)	12	(7.7)	**0.001**
Out-of-hospital	0	(0.0)	6	(3.9)	**0.001**
In-hospital	4	(1.2)	6	(3.9)	0.055
Stroke	5	(1.5)	11	(7.1)	**0.001**
**Medication on admission**, n (%)					
ACE-inhibitor	138	(41.9)	52	(33.5)	0.078
ARB	86	(26.1)	31	(20.0)	0.141
Beta-blocker	234	(71.1)	98	(63.2)	0.081
Aldosterone antagonist	47	(14.3)	14	(9.0)	0.104
ARNI	3	(0.9)	2	(1.3)	0.701
SGLT2-inhibitor	7	(2.1)	2	(1.3)	0.525
Loop diuretics	230	(69.9)	68	(43.9)	**0.001**
Statin	169	(51.4)	70	(45.2)	0.203
ASA	93	(28.3)	59	(38.1)	**0.030**
P2Y12-inhibitor	36	(10.9)	13	(8.4)	0.385
DOAC	124	(37.7)	39	(25.2)	**0.007**
Vitamin K antagonist	37	(11.2)	13	(8.4)	0.335

Level of significance p ≤ 0.05. Bold type indicates statistical significance

*ACE* angiotensin-converting-enzyme, *ARB* angiotensin receptor blocker, *ARNI* angiotensin receptor neprilysin inhibitor, *ASA* acetylsalicylic acid, *CABG* coronary artery bypass grafting, *CKD* chronic kidney disease, *COPD* chronic obstructive pulmonary disease, *CRT-D* cardiac resynchronization therapy with defibrillator, *DBP* diastolic blood pressure, *DOAC* directly acting oral anticoagulant, *HFmrEF* heart failure with mildly reduced ejection fraction, *HFpEF* heart failure with preserved ejection fraction, *HFrEF* heart failure with reduced ejection fraction, *IQR* interquartile range, *LVEF* left ventricular ejection fraction, *(N)STEMI* non-ST-segment elevation myocardial infarction, *SBP* systolic blood pressure, *SGLT2* sodium glucose linked transporter 2, *(s) ICD* (subcutaneous) implantable cardioverter defibrillator

**Table 2:** Heart-failure related and procedural data

	Primary ADHF (*n* = 329)		Secondary ADHF (*n* = 155)		p value
Heart failure etiology, n (%)					
Ischemic cardiomyopathy	181	(55.0)	100	(64.5)	
Non-ischemic cardiomyopathy	20	(6.1)	11	(7.1)	
Hypertensive cardiomyopathy	26	(7.9)	5	(3.2)	
Congenital heart disease	1	(0.3)	0	(0.0)	
Valvular heart disease	30	(9.1)	6	(3.9)	0.058
Tachycardia associated	20	(6.1)	9	(5.8)	
Tachymyopathy	10	(3.0)	2	(1.3)	
Pacemaker-induced cardiomyopathy	6	(1.8)	0	(0.0)	
Unknown	35	(10.6)	22	(14.2)	
NYHA functional class, n (%)					
I/II	71	(21.6)	60	(38.7)	
III	151	(45.9)	54	(34.8)	**0.001**
IV	107	(32.5)	41	(26.5)	
Echocardiographic data					
LVEF, %, median (IQR)	45	(45–47)	45	(44–47)	0.857
IVSd, median (IQR)	12	(11–14)	12	(10–13)	**0.007**
LVEDD, mm, median (IQR)	50	(46–55)	47	(43–52)	**0.001**
TAPSE, mm, median (IQR)	20	(16–22)	19	(16–23)	0.769
LA diameter, mm, median (IQR)	46	(40–51)	44	(37–47)	**0.005**
LA surface, cm^2^, median (IQR)	25	(21–30)	23	(20–27)	**0.015**
E/A, median (IQR)	1.0	(0.7–1.6)	0.9	(0.6–1.3)	0.137
E/E`, median (IQR)	12.7	(7.5–18.5)	12.0	(7.0–16.0)	0.142
Diastolic dysfunction, n (%)	249	(75.7)	110	(71.0)	0.269
Moderate-severe aortic stenosis, n (%)	48	(14.6)	25	(16.1)	0.659
Moderate-severe aortic regurgitation, n (%)	28	(8.5)	11	(7.1)	0.594
Moderate-severe mitral regurgitation, n (%)	80	(24.3)	36	(23.2)	0.793
Moderate-severe tricuspid regurgitation, n (%)	99	(30.1)	52	(33.5)	0.444
TR Vmax, m/s, median (IQR)	3.10	(2.70–3.40)	3.00	(2.70–3.40)	0.889
VCI, mm, median (IQR)	24	(19–28)	22	(18–26)	0.386
Aortic root, mm, median (IQR)	32	(29–35)	33	(28–36)	0.614
Coronary angiography, n (%)	114	(34.7)	67	(43.2)	0.069
No evidence of coronary artery disease	32	(28.1)	10	(14.9)	
1-vessel disease	24	(21.1)	8	(11.9)	
2-vessel disease	15	(13.2)	5	(7.5)	**0.004**
3-vessel disease	43	(37.7)	44	(65.7)	
CABG	10	(8.8)	5	(7.5)	0.758
Chronic total occlusion	13	(11.4)	8	(11.9)	0.913
PCI, n (%)	39	(34.2)	39	(58.2)	**0.002**
Sent to CABG, n (%)	6	(5.3)	6	(9.0)	0.335
Baseline laboratory values, median (IQR)					
Potassium, mmol/L	3.8	(3.5–4.2)	3.8	(3.5–4.2)	0.537
Sodium, mmol/L	139	(137–142)	139	(137–142)	0.991
Creatinine, mg/dL	1.40	(1.04–1.98)	1.12	(0.87–1.88)	**0.005**
eGFR, mL/min/1.73 ^2^	46	(31–65)	55	(30–79)	**0.006**
Hemoglobin, g/dL	11.4	(9.7–13.2)	10.6	(9.3–11.8)	**0.001**
WBC count, × 10^9^/L	8.08	(6.35–10.21)	8.77	(6.64–11.81)	**0.034**
Platelet count, × 10^9^/L	233	(175–288)	231	(176–295)	0.499
HbA1c, %	6.2	(5.7–7.6)	6.0	(5.5–6.9)	0.175
LDL-cholesterol, mg/dL	86	(62–112)	85	(66–119)	0.488
HDL-cholesterol, mg/dL	42	(35–54)	40	(29–47)	**0.014**
C-reactive protein, mg/L	18	(6–47)	38	(15–91)	**0.001**
NT-proBNP, pg/mL	5164	(2377–10,102)	7644	(2812–16,506)	**0.024**
NT-proBNP (eGFR corrected), pg/mL	2311	(1275–4196)	3608	(1605–8091)	**0.002**
Cardiac troponin I, µg/L	0.03	(0.02–0.10)	0.12	(0.02–1.36)	**0.001**
Medication at discharge, n (%)					
ACE-inhibitor	147	(46.8)	66	(47.8)	0.843
ARB	96	(30.6)	24	(17.4)	**0.003**
Beta-blocker	265	(84.4)	120	(87.0)	0.480
Aldosterone antagonist	79	(25.2)	25	(18.1)	0.101
ARNI	3	(1.0)	4	(2.9)	0.123
SGLT2-inhibitor	15	(4.8)	2	(1.4)	0.087
Loop diuretics	292	(93.0)	115	(83.3)	**0.002**
Statin	200	(63.7)	82	(59.4)	0.388
Digitalis	22	(7.0)	7	(5.1)	0.440
Amiodarone	14	(4.5)	5	(3.6)	0.684
ASA	111	(35.4)	73	(52.9)	**0.001**
P2Y12-inhibitor	74	(23.6)	50	(36.2)	**0.005**
DOAC	150	(47.8)	49	(35.5)	**0.016**
Vitamin K antagonist	33	(10.5)	8	(5.8)	0.108

*ACE* angiotensin-converting enzyme, *ARB* angiotensin receptor blocker, *ARNI* angiotensin receptor neprilysin inhibitor, *ASA* acetylsalicylic acid, *CABG* coronary artery bypass grafting, *COPD* chronic obstructive pulmonary disease, *DOAC* directly acting oral anticoagulant, *eGFR* estimated glomerular filtration rate, *HbA1c* glycated haemoglobin, *HDL* high-density lipoprotein, *IQR* interquartile range, *IVSd* Interventricular septal end diastole, *LA* left atrial, *LDL* low-density lipoprotein, *LVEDD* Left ventricular end-diastolic diameter, *LVEF* left ventricular ejection fraction, *NT-pro BNP* aminoterminal pro-B-type natriuretic peptide, *NYHA* New York Heart Association, *PCI* percutaneous coronary intervention, *SGLT2* sodium glucose linked transporter 2, *TAPSE* tricuspid annular plane systolic excursion, *VCI* Vena cava inferior, *WBC* white blood cells. Level of significance p ≤ 0.05. Bold type indicates statistical significance

